# Antiepileptic Potential of Matrine via Regulation the Levels of Gamma-Aminobutyric Acid and Glutamic Acid in the Brain

**DOI:** 10.3390/ijms141223751

**Published:** 2013-12-05

**Authors:** Jun Xiang, Yugang Jiang

**Affiliations:** Department of Neurosurgery, Second Xiangya Hospital, Central South University, Changsha 410011, China; E-Mail: xiangjunxy@126.com

**Keywords:** antiepileptic, matrine, gamma-aminobutyric acid, glutamic acid

## Abstract

Our present study aimed to determine the antiepileptic activity of matrine, and explore the possible molecular mechanism. To evaluate the antiepileptic activity of matrine, seizures in mice induced by PTZ and MES were established, then the pentobarbital sodium-induced anaesthetizing time and locomotor activity tests in mice were also carried out. For the molecular mechanism investigations, contents of aspartic acid (Asp), gamma-aminobutyric acid (GABA), glutamic acid (Glu), glycine (Gly) in seizures mice were determined; then, the chronic seizures rats induced by PTZ were prepared, and western blotting was used to determine the expressions of GAD 65, GABA_A_ and GABA_B_ in the brains. In the results, matrine showed significant antiepileptic effects on seizures mice induced by MES and PTZ. Moreover, the pentobarbital sodium-induced anaesthetizing time and locomotor activity tests were also demonstrated that matrine had obvious antiepileptic effects. Additionally, our results revealed that after treatment with matrine, contents of GABA can be elevated, and the contents of Glu were obviously decreased. Furthermore, western blotting revealed that the mechanism regarding the antiepileptic effect of may be related to the up-regulations of GAD 65 and GABA_A_ in the brain. Collectively, we suggested that matrine can be developed as an effective antiseptic drug.

## 1. Introduction

Epilepsy, a common chronic neurological disorder, is characterized by recurrent unprovoked seizures, leading to suffering in an estimated 50 million people worldwide [1,2]. Epilepsy is the result of complex reasons and can cause convulsions, sensory disturbances, or consciousness [3]. In spite of great improvements that have been made in diagnosis and treatment of epilepsy, epilepsy is still a devastating and poorly treated disease, which can require lengthy and costly treatments to manage [2,4]. Currently, almost all antiepileptic drugs used have obvious safety and efficacy deficits, and, therefore, patients are often reluctant to take these drugs [5]. Therefore, it is necessary to discover new and reliable therapeutic agents with few side effects for treating epilepsy.

**Figure 1.**
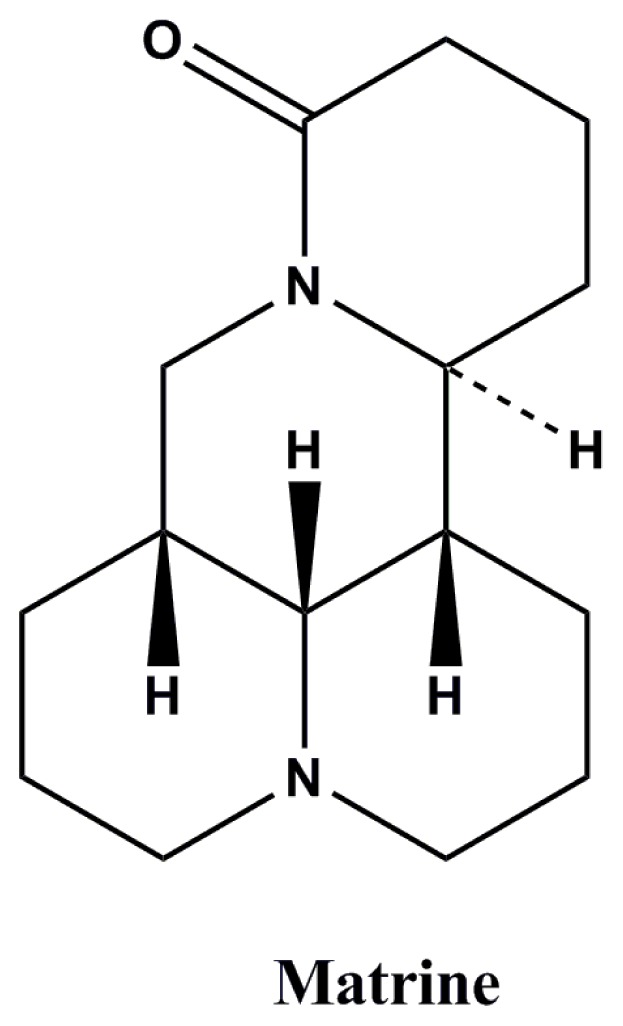
Structure of matrine.

Plant-derived natural extracts or compounds have been used in folk medicine for thousands years for treating various diseases. Currently, more and more attention has been paid to plant-derived medicines [6,7]. Matrine (Figure 1) is the major alkaloid of *Sophora flavescens* which belongs to the family of *Leguminosae*, and has been reported to have lots of physiological activities, including anti-bacterial, anti-inflammatory, anti-tumor, anti-oxidant, and anti-ulcerative [8,9]. Matrine has a wide spectrum of pharmacological activities, like *S. flavescens*, such as anti-cerebral ischemia, protection of neurons, anti-nociceptive, anti-inflammatory, and anti-tumor, *etc.* [8,9]. However, to the best of our knowledge, there is no report on the antiepileptic effect of the matrine. As a part of our systematic search for new antiepileptic agents from plants, the matrine was found to have significant antiepileptic effect in our preliminary experiment. The present study aimed to systemically investigate the antiepileptic effects of matrine in mice and rats, which has significant reference value for using matrine to treat epilepsy in clinics.

## 2. Results and Discussion

### 2.1. Result of the Maximal Electroshock Test

As shown in Table 1, the matrine at 7.5 mg/kg had no protective effect against maximal electroshock-induced seizures. However, the matrine at the tested doses of 15, 30 and 60 mg/kg exhibited significant antiepileptic effects (*p* < 0.05, *p* < 0.01, *p* < 0.01, respectively). In addition, the percentages of tonic clonic seizures at the tested doses of matrine (7.5, 15, 30 and 60 mg/kg) were 73.3%, 40%, 20% and 0%, respectively (*p* < 0.01, *p* < 0.01, *p* < 0.01 for the doses of 15, 30 and 60 mg/kg, compared to the control group, respectively).

Phenytoin was used as a positive control. Mice were divided into six groups (*n* = 15): control group (mice were treated with normal saline, 20 mL/kg, ip), positive group (mice were treated with phenytoin, 20 mg/kg, ip), and four tested matrine groups (mice were treated with matrine at the doses of 7.5, 15, 30 and 60 mg/kg, ip). The value of the “Convulsions” and “Death” columns were number of the mice. The Chi-squared exact test was used to analyze significance of anticonvulsant effects against MES-induced seizures among groups (Inhibition/% and Mortality/%), *p < 0.05, **p < 0.01, *vs.* control group.

### 2.2. Result of Pentylenetetrazole-Induced Seizure Test

To confirm the antiepileptic effects of matrine, the PTZ-induced seizures mice model was established to evaluate the protective effects against seizures. From the results of our present study, matrine at the tested doses of 7.5, 15, 30 and 60 mg/kg showed the obvious antiepileptic effects (*p* < 0.05, *p* < 0.01, *p* < 0.01, *p* < 0.01, respectively) (Table 2). Furthermore, the matrine at all the tested doses of 7.5, 15, 30 and 60 mg/kg can significantly decrease mortality (mortality was 60%, 40%, 26.6% and 13.3%, respectively; *p* < 0.05, *p* < 0.05, *p* < 0.01 and *p* < 0.01, respectively).

Diazepam was used as a positive control. Mice were divided into six groups (*n* = 15): control group (mice were treated with normal saline, 20 mL/kg, ip), positive group (mice were treated with diazepam, 4 mg/kg, ip), and four tested matrine groups (mice were treated with matrine at the doses of 7.5, 15, 30 and 60 mg/kg, ip). The values of the “Convulsions” and “Death” columns were the number of the mice. The Chi-squared exact test was used to analyze the significance of anticonvulsant effects against MES-induced seizures among groups (Inhibition/% and Mortality/%), *p < 0.05, **p < 0.01, *vs.* control group.

### 2.3. Result of Pentobarbital Sodium-Induced Anaesthetizing Time in Mice

**Figure 2.**
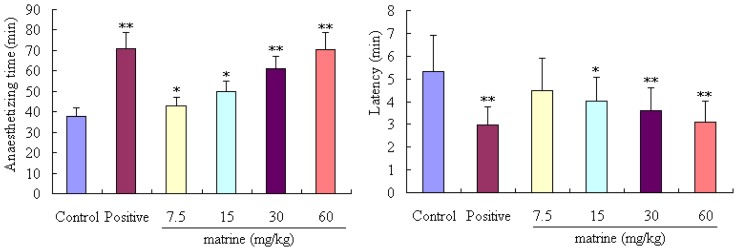
Effects of matrine on pentobarbital sodium-induced anaesthetizing time in mice. Diazepam was used as a positive control. Mice were divided into six groups (*n* = 10): control group (mice were treated with normal saline, 20 mL/kg, ip), positive group (mice were treated with diazepam, 4 mg/kg, ip), and four tested matrine groups (mice were treated with matrine at the doses of 7.5, 15, 30 and 60 mg/kg, ip). Values are means ± SD (*n* = 10), the two-tailed Student’s *t* test was used to compared the difference among groups, * *p* < 0.05, ** *p* < 0.01, *vs.* control group.

As can be seen from Figure 2, the duration of anaesthetizing time can be prolonged by treatment with matrine at doses of 7.5, 15, 30 and 60 mg/kg (*p* < 0.05, *p* < 0.05, *p* < 0.01 and *p* < 0.01, respectively), and the sleep latencies can be also decreased by treating with matrine at the doses of 15, 30 and 60 mg/kg (*p* < 0.05, *p* < 0.05 and *p* < 0.01).

### 2.4. Results of Mouse Locomotor Activity Tests

**Figure 3.**
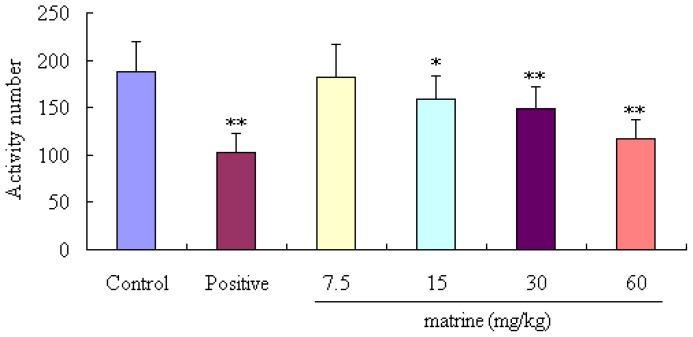
Effects of matrine on locomotor activity in mice. Diazepam was used as a positive control. Mice were divided into six groups (*n* = 10): control group (mice were treated with normal saline, 20 mL/kg, ip), positive group (mice were treated with diazepam, 4 mg/kg, ip), and four tested matrine groups (mice were treated with matrine at the doses of 7.5, 15, 30 and 60 mg/kg, ip). Values are means ± SD (*n* = 10), the two-tailed Student’s *t* test was used to compared the difference among groups, * *p* < 0.05, ** *p* < 0.01, *vs.* control group.

Results of locomotor activity test were shown in Figure 3, matrine can significant decrease the locomotor activity of mice at the doses of 15, 30 and 60 mg/kg (*p* < 0.05, *p* < 0.01 and *p* < 0.01, respectively) compared to control animals.

### 2.5. Results of the Contents of Asp, GABA, Glu, and Gly in Mouse Brain

**Figure 4.**
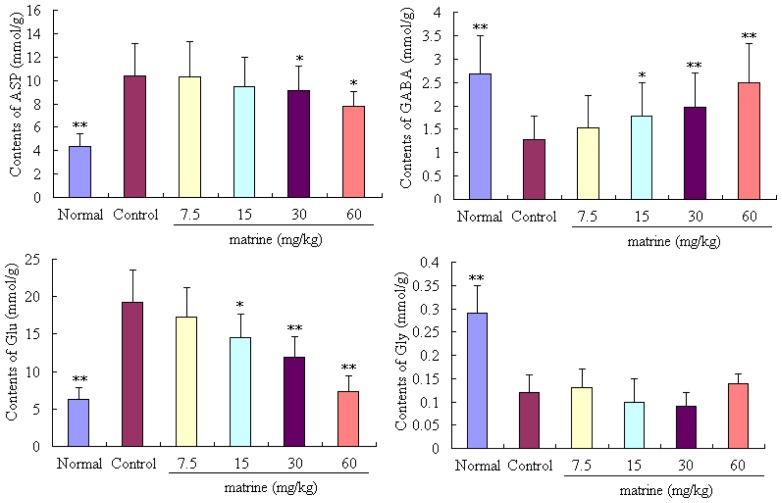
Effects of matrine on aspartic acid (ASP), gamma-aminobutyric acid (GABA), glutamic acid (Glu) and glycine (Gly) in the mouse brain. Mice were divided into six groups (*n* = 10): normal group (mice were not suffered PTZ, and treated with normal saline, 20 mL/kg, ip), control group (mice were treated with normal saline, 20 mL/kg, ip), and four tested matrine groups (mice were treated with matrine at the doses of 7.5, 15, 30 and 60 mg/kg, ip). Values are means ± SD (*n* = 10), the two-tailed Student’s *t* test was used to compared the difference among groups, * *p* < 0.05, ** *p* < 0.01, *vs.* control group.

Matrine can significantly decrease the brain Asp at the doses of 30 and 60 mg/kg in mice with seizures induced by PTZ, compared to the control group (*p* < 0.05, *p* < 0.05, respectively). In addition, Glu can be significantly decreased in a dose-dependent manner by treatment with matrine at 15, 30 and 60 mg/kg (*p* < 0.05, *p* < 0.01, and *p* < 0.01, respectively). In contrast, the contents of GABA can be increased in mice with seizures by treatment with matrine at the doses of 15, 30 and 60 mg/kg (*p* < 0.05, *p* < 0.01, *p* < 0.01, respectively), in a dose-dependent manner. However, no obvious difference was observed in all the tested doses of matrine, compared to the control group (*p* > 0.05) (Figure 4).

### 2.6. Effect of Matrine on Expressions of GAD 65, GABA_A_, GABA_B_ in the Brain of Epileptic Rats

**Figure 5.**
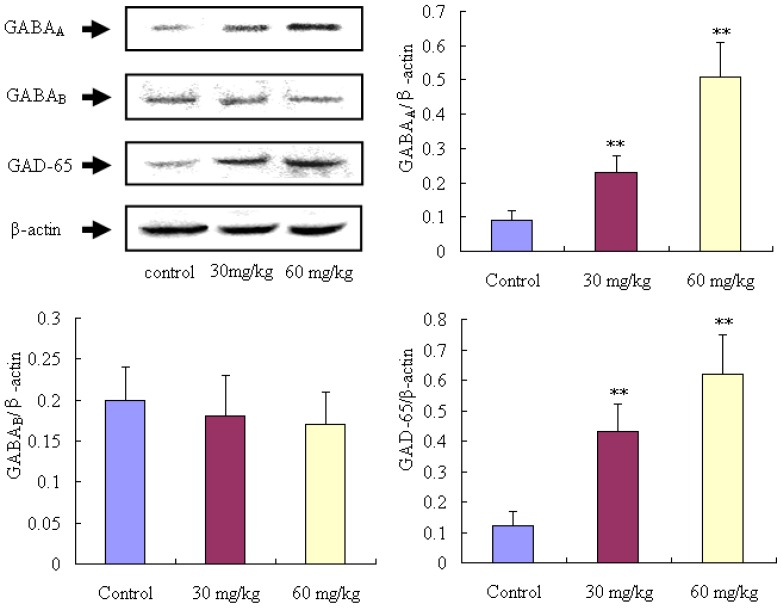
Effects of matrine on GAD 65, GABA_A_, GABA_B_ expressions in the brain of epileptic rats. Mice were divided into 3 groups (*n* = 10): control group (mice were treated with normal saline, 20 mL/kg, i.p), and 2 tested matrine groups (mice were treated with matrine at the doses of 30 and 60 mg/kg, i.p). Values are means ± SD (*n* = 10), the two-tailed Student’s *t* test was used to compared the difference among groups, **p < 0.01, *vs.* control group.

Our results are shown in the Figure 5. GAD65 and GABA_A_ expressions in the mice brains after treatment with matrine were significantly up-regulated (*p* < 0.01) compared to the control group animals. However, no obvious difference was observed in the expression of GABA_B_ between the matrine treated rats and control rats (*p* > 0.05).

### 2.7. Discussion

To the best of our knowledge, this is the first systemic report regarding antiepileptic effect of matrine, and the mechanism may be related to regulate the levels of GABA and Glu in brain. Currently, drugs for treatment of epilepsy are limited due to the effects of present drugs are not very good and/or they have severe side-effects [5]. Previous investigation reported that matrine was a safe agent with the LD_50_ over 80 mg/kg by intravenous, and had a LD_50_ over 150 mg/kg by intraperitoneal injection [10]. Seizures model induced by PTZ and MES were the most commonly used preliminary screening tests for finding potential antiepileptic drugs in laboratory [11]. The MES test is the model for evaluating antiepileptic effect against generalized tonic-clonic seizures. However, the PTZ test is considered to be a valid model for human generalized myoclonic and absence seizures [1,11]. In our present study, our results revealed that the matrine can block both clonic seizures induced by PTZ and tonic seizures induced by MES. Moreover, the locomotor activity and pentobarbital sodium-induced anaesthetizing time tests in mice, which are two commonly used and reliable ways to study sedative activity of drugs [12,13], were performed, and the results also demonstrated that the matrine showed significant effect. Collectively, our present investigation can demonstrate that matrine had significant antiepileptic effects.

GABA is the important and principal inhibitory neurotransmitters in the CNS, and plays a key role in epilepsy disease. In contrast, the Glu is the important excitatory neurotransmitters in the brain, which is also closely related to the development of epilepsy. Moreover, considerable previous investigations have demonstrated that the imbalance of GABA/Glu in the brain is a very important factor in epilepsy [14,15]. In our present study, the results revealed that, after treatment with matrine, the contents of GABA and Gly (another important inhibitory neurotransmitter in brain) can be elevated, and, however, the content of Glu was decreased. For further molecular mechanism investigation, the expressions of Glutamic acid decarboxylase 65 (GAD 65), GABA_A_, and GABA_B_ were determined. GAD is the key rete-limiting enzyme for synthesis of GABA, and the GABA_A_, and GABA_B_ are the receptors of GABA, which are the primary target for development of anticonvulsant drugs [13]. Abundant reports demonstrated that the expressions of GAD and GABA receptors in the brain are closely correlated with epilepsy [1,14–16]. In our present study, we demonstrated that the expressions of GAD 65 and GABA_A_ can be up-regulated in the brain of epileptic mice by treatment with matrine, which is a potential molecular mechanism of matrine for treatment of epilepsy.

## 3. Experimental Section

### 3.1. Animals

Animals were purchased from the Shanghai laboratory animal center (Shanghai, China). Male ICR mice (20 ± 2 g) or SD rats (200 ± 20 g) were used in our study. Animals were kept on a 12 h light/dark cycle with free access to standard laboratory chow and water. Humidity was maintained at 50% and the temperature at 25 °C. Each animal was used only once in the experiment. The experimental protocols were approved by the Animal Care and Use Committee of our hospital and complied with the National Institutes of Health Guide for care and Use of Laboratory Animals.

### 3.2. Drugs and Chemicals

Matrine, pentylenetetrazole, phenytoin sodium, and pentobarbital sodium were purchased from the Aladdin Reagent Inc. (Shanghai, China). Diazepam, the standard agents of aspartic acid (Asp), gamma-aminobutyric acid (GABA), glutamic acid (Glu), glycine (Gly) were purchased from the National Institutes for Food and Drug Control (Beijing, China). GAD-65, GABA_A_, and GABA_B_ monoclonal antibody were purchased from Abcam Co. (Hong Kong, China). All other chemicals used in this study were of analytical reagent grade.

### 3.3. Protocols

The antiepileptic activity of matrine was evaluated by maximal electroshock and pentylenetetrazole-induced seizures, pentobarbital sodium-induced sleeping time and locomotor activity tests in mice. Finally, the mechanism of the antiepileptic activity of matrine was explored by evaluating the contents of Asp, GABA, Glu, and Gly in animal brains, and Western blot was used to measure GAD65, GABA_A_, and GABA_B_ expressions in animal brains. The positive control dosage was based on known pharmacokinetics and previous clinical use. Thus, matrine was administered intraperitoneally and the dose selections of 7.5, 15, 30 and 60 mg/kg were based on the results of preliminary experiments.

### 3.4. Maximal Electroshock (MES)-Induced Seizures in Mice

Maximal electroshock seizure (MES) test was carried out according to the previously reported methods with some modifications [17]: an alternating current stimulus (50 mA; 50 Hz; 1 s duration) was used. Mice were pretreated with matrine (7.5, 15, 30 and 60 mg/kg), phenytoin (20 mg/kg), and normal saline (20 mL/kg) by intraperitoneal injection (ip), and after 30 min, electroshock was administered. Mice hind limb tonic extension (HLTE) was observed within 10 s after electroshock delivery, and the criterion for the anticonvulsant effect was the absence of an HLTE and the later tonic-clonic seizures.

### 3.5. Pentylenetetrazole (PTZ)-Induced Convulsion in Mice

The pentylenetetrazole (PTZ)-induced convulsion test was performed according to the previously described with some modifications [18,19], and used to induce convulsions in mice with pentylenetetrazole (PTZ). Mice were pretreated with matrine (7.5, 15, 30 and 60 mg/kg), diazepam (4 mg/kg, ip), and normal saline (20 mL/kg, ip). 30 min after treatment, mice in all groups were administered PTZ (90 mg/kg) subcutaneously (sc). Mice were observed within 60 min, and 5 different types of seizure stages were divided by the criterions of Racine’s scale [17,18], including: (stage 1) one or more mouth and facial twitches; (stage 2) rhythmicity head nodding; (stage 3) forelimb clonus; (stage 4) seizures characterized by rearing or hind limb extension; (stage 5) seizures with rearing and repeated falling.

### 3.6. Pentobarbital Sodium-Induced Anaesthetizing Time in Mice

The pentobarbital-induced anaesthetizing time in mice was determined according to the previously reported with some modifications [20]. Mice were pretreated with matrine (7.5, 15, 30 and 60 mg/kg, ip), diazepam (4 mg/kg, ip), or normal saline (20 mL/kg, ip) and 30 min before injection of pentobarbital sodium (50 mg/kg, ip). Mice were observed for the loss of the “righting reflex”—The mouse rolls over onto its feet when turned on its back. The time between the loss and recovery of the righting reflex was recorded as the duration of sleep.

### 3.7. Measurement of Locomotor Activity

Locomotor activity determination was performed according to the method described previously [15] with some modifications. Mice were pretreated with matrine (7.5, 15, 30 and 60 mg/kg, ip), diazepam (4 mg/kg, ip), and normal saline (20 mL/kg, ip), there were 10 mice in each group. A photoelectrical spontaneous locomotor activity apparatus was used (size of the open field was 60 cm × 60 cm × 40 cm). In the experiment, a movement of the mice could interrupt the beam of light falling on the photocell of the apparatus, then a count was recorded. Each mouse was placed individually in the center of the apparatus for 5 min and the numbers of activities of mice were auto-recorded during a period of 5 min by the apparatus.

### 3.8. Measurement of the Contents of Asp, GABA, Glu, and Gly in Brain

PTZ-induced convulsions in mice were prepared as described above. Mice were pretreated with matrine (7.5, 15, 30 and 60 mg/kg, ip), and normal saline (20 mL/kg, ip). Mice were sacrificed 50 min after they received PTZ or saline, and the whole brain was collected from each animal. Then, brain tissues were homogenized and weighed and then the contents of Asp, GABA, Glu, and Gly in brain were determined by HPLC method.

### 3.9. Chronic Epileptic Rat Seizure Induced by PTZ and Western Blotting

The chronic epileptic rat model was prepared according to the method described previously [20]. 30 SD rats were divided into three groups: control group, and two matrine treatment groups (30 and 60 mg/kg). PTZ (40 mg/kg, ip) was administered once/day for 4 weeks. The criterion for the evaluation of the chronic epileptic model was “strong jerking and jumping, and shouting”. After the chronic epileptic model of rats was established, rats were pretreated with normal saline (20 mL/kg, ip) and matrine (30 and 60 mg/kg/day, ip) for 21 days. Then, rats were sacrificed after anesthesia, and whole brains were collected. Total brain tissue protein was extracted, and equal amounts of protein (40 μg) were separated by SDS-PAGE, blotted on PVDF membranes, and probed with anti-GAD 65, GABA_A_, GABA_B_ rabbit polyclonal IgG and subsequently with goat anti-rabbit/HRP, and detected with chemiluminescence. To measure protein loading, antibodies directed against β-actin were used.

### 3.10. Statistical Analysis

The Chi-squared exact test was used to analyze significance of anticonvulsant effects against MES and PTZ-induced seizures among groups (Inhibition/% and Mortality/%). All other data are presented as mean ± standard deviation, and statistical analyses were performed using the two-tailed Student’s *t* test with a significance level of *p* < 0.05.

## 4. Conclusions

In conclusion, matrine has significant antiepileptic effect in mice and rats, and the contents of GABA and Glu in the brain can be regulated by treatment with matrine. Moreover, the molecular mechanism may be related to the up-regulation of GABA_A_ receptor and GAD 65 expressions in the brain. Therefore, the matrine can be developed as an effective drug for treating epilepsy in the future.

## Figures and Tables

**Table 1.** Effect of matrine on tonic seizures induced by maximal electroshock in male mice.

Treatment	Dose	Convulsions (*n*)	Inhibition (%)	Tonic-clonus (%)	Death (*n*)	Mortality (%)
Control	20 mL/kg	15	0	100	3	20

Positive	20 mL/kg	0	100 **	0 **	0	0

Matrine	7.5 mg/kg	12	20	73.3	2	13.3
	15 mg/kg	8	46.7 *	40 **	0	0
	30 mg/kg	4	73.3 **	20 **	0	0
	60 mg/kg	0	100 **	0 **	0	0

* *p* < 0.05,

** *p* < 0.01, *vs.* control group.

**Table 2.** Effect of matrine on pentylenetetrazole (PTZ)-induced seizures in male mice.

Treatment	Dose	Convulsions (*n*)		Inhibition (%)		Death (*n*)	Mortality (%)
	
		IV–V	V	IV–V	V		
Control	20 mL/kg	15	15	0	0	15	100

Positive	4 mL/kg	0	0	100 **	100 **	0	0 **

Matrine	7.5 mg/kg	8	7	46.7 *	53.3 *	9	60 *
	15 mg/kg	7	5	53.3 **	66.7 **	6	40 *
	30 mg/kg	5	3	66.7 **	80 **	4	26.6 **
	60 mg/kg	4	2	73.3 **	86.6 **	2	13.3 **

* *p* < 0.05,

** *p* < 0.01, *vs.* control group.
